# Predictors of high and low mental well-being and common mental disorders: findings from a Danish population-based study

**DOI:** 10.1093/eurpub/ckaa021

**Published:** 2020-02-27

**Authors:** Ziggi Ivan Santini, Sarah Stougaard, Ai Koyanagi, Annette Kjær Ersbøll, Line Nielsen, Carsten Hinrichsen, Katrine R Madsen, Charlotte Meilstrup, Sarah Stewart-Brown, Vibeke Koushede

**Affiliations:** c1 The National Institute of Public Health, University of Southern Denmark, Copenhagen, Denmark; c2 University of Southern Denmark, Odense, Denmark; c3 Parc Sanitari Sant Joan de Déu, Universitat de Barcelona, Fundació Sant Joan de Déu, CIBERSAM, Barcelona, Spain; c4 ICREA, Barcelona, Spain; c5 Division of Health Sciences, Warwick Medical School, University of Warwick, Coventry, UK

## Abstract

**Background:**

Mental well-being is fundamental for a good life. Previous literature has examined the predictors of mental disorders and continuous measures of positive mental health. Very few studies have specifically focused on the predictors of different levels of mental well-being, but those that have suggest a different picture. This study aimed to compare socioeconomic and relational/recreational behaviour predictors of different levels of mental well-being as well as common mental disorders (CMDs).

**Methods:**

Data from 3508 adults aged 16+ years old from the Danish Mental Health and Well-Being Survey 2016 were linked to Danish national register-based data. Mental well-being was assessed using the Warwick-Edinburgh Mental Well-being Scale, and information on CMDs was assessed using the Patient Health Questionnaire (PHQ-4). Regression analyses were conducted to estimate the predictors of low and high mental well-being compared to moderate mental well-being and also of CMDs.

**Results:**

Lower socioeconomic position (education, income and employment status) was associated with increased odds of low mental well-being and the presence of CMDs, but did not significantly predict high mental well-being. Relational/recreational behaviours (informal and formal social participation, social support and recreational activity) were associated with reduced odds of low mental well-being and CMDs, and also with increased odds of high mental well-being.

**Conclusions:**

Socioeconomic predictors of high mental well-being do not mirror those of low mental well-being and CMDs, whereas relational/recreational predictors of high mental well-being do mirror those of low mental well-being and CMDs. These findings have important implications for public mental health strategies.

## Introduction

Acknowledgement of the importance of positive mental health and the promotion of mental well-being for public health is growing among researchers and policymakers, partly as a response to the growing burden of mental disorders globally.^1^^,^^2^ Mental well-being is a relatively new concept defined in different ways in different disciplines.^3^ Some disciplines favour the hedonic aspects (positive feelings, affect, emotions and life satisfaction) also referred to as subjective well-being, and some favour the eudaimonic aspects (positive functioning, mindset and relationships) also referred to as psychological well-being,^3^^,^^4^ but these distinctions are not fixed. In the context of public health in the UK, mental well-being is defined as both hedonic (feeling good) and eudaimonic (functioning well) and the two are regarded as integral to one another.^5^ Functioning well includes living in a way that brings meaning and purpose, a point which often seems to be neglected within mental health research. Mental disorders are diagnosed on the basis of not feeling well and functioning poorly, positioning the presence of mental well-being (higher levels), as defined here, at one end of a single continuum, with mental illness or the absence of mental well-being (lower levels) at the opposite end. This also implies strong correlations between continuous measures of mental illness and mental well-being.^4^ Enhancing population levels of positive mental health is important from a public health standpoint for several reasons: it is desirable in its own right,^6^ it is economically worthwhile,^7^^,^^8^ it can prevent mental disorders and somatic illness, and promote recovery in individuals with mental health problems.^9–11^

Most public mental health policy focuses on the prevention of mental disorders,^12^ particularly highly prevalent common mental disorders (CMDs) e.g. depression and anxiety, which have major impacts on the burden of disease.^1^ Yet, improving mental well-being in individuals with and without mental disorders is fundamental to sustaining population mental health as well as reducing the burden of mental disorders.^5^ In order to promote population-level mental health, it is necessary to explore the predictors of different levels of mental well-being. Otherwise, strategies may be based on a ‘one size fit all’ approach that may not achieve the desired outcomes.

From a ‘hierarchy of needs’ theoretical perspective, humans seek to fulfil their needs in a hierarchical manner.^13^ According to this perspective, improvements in material conditions will lead to increases in well-being when basic needs (physiological and safety needs) are dominant. This does not imply that higher needs at this point do not affect well-being, but rather that basic needs take precedence. When basic needs are satisfied and needs shift up the hierarchy, the utility of improved material conditions will attenuate, while the satisfaction of higher needs (e.g. love/belonging, self-actualization) will continue to lead to increases in well-being. Accordingly, research has found that well-being increases proportionately with economic conditions up to a certain point, after which the rise in well-being attenuates with continuing increases in economic conditions.^14^ Whilst economic conditions are considered to pertain mainly to basic physiological/safety needs, relational needs—such as needs for love and belonging—are generally illustrated as being positioned higher up in the hierarchy. They nevertheless do not pertain strictly to higher needs. The need for social connectedness is a deeply ingrained human characteristic that has evolved hand in hand with neural, hormonal and genetic mechanisms directly associated with bonding, companionship and herd behaviour, as a crucial means for ensuring survival and reproduction. Relational needs therefore also pertain to very basic needs, such as physiological and safety needs.^15^ This implies that relational factors may affect well-being at all levels.

A few studies have suggested that socioeconomic predictors of low mental well-being are similar to those of mental disorders, but that these socioeconomic factors do not play the same role in predicting high mental well-being.^16–18^ However, other studies have shown that relational factors (e.g. greater social connectedness and support) and recreational activities are both associated with reduced risks for mental disorders, as well as being positively associated with outcomes pertaining to positive mental health.^19^^,^^20^

Specifically, Stewart-Brown et al.^16^ conducted a study on a representative sample of the adult English population and found evidence confirming the expected strong association between low socioeconomic position and low mental well-being, but no association between these factors and high mental well-being. Van Lente et al.^18^ did not compare predictors of high and low mental well-being, but compared the predictors of positive and negative mental health states in Ireland and found that social well-being positively predicted high levels of both positive and negative mental health, while socioeconomic factors predicted negative mental health states only. Nielsen et al.^17^ investigated whether this pattern also applied to Danish adolescents and found an association between low socioeconomic position and indicators of low mental well-being, but no association between socioeconomic position and indicators of high mental well-being. A study in elderly populations in Finland found a similar discrepancy between educational level and employment as a predictor of low and high mental well-being.^21^

Relational and recreational factors are essential when assessing predictors of mental health and well-being. Factors pertaining to social connectedness, such as social interaction and support, are thought to be related to mental health via a number of biological (e.g. physiological processes, immune function, arousal), psychological (e.g. behavioural guidance, social comparison and influence, affective states) and social pathways (e.g. social capital, bonding and belongingness), and may promote resilience and serve as a buffer against stress.^22^ Social participation, such as volunteering, community/social group activity and helping behaviour, is thought to contribute to enhanced mental health by: (i) providing a sense of meaning and purpose through contribution to the community, (ii) providing psychological and cultural resources, such as competences, skills and values and (iii) providing opportunities for social connectedness.^23^ Recreational activities, such as taking on and meeting challenges, even small ones, is thought to provide feelings of efficacy and a stronger sense of self, which have implications for self-esteem, competence, motivation and feelings of control.^24^^,^^25^ Being engaged with an activity and focusing attention on it (as opposed to focusing on things not going on at the present moment) has been found to predict well-being.^26^

Recent studies investigating the relationship of relational and recreational factors to mental health status in populations, however, appear to find a mirroring association between predictors of high and low mental well-being. In elderly populations in Finland, social support was strongly associated high mental well-being and lack of it strongly associated with low mental well-being.^21^ Other studies have shown that relational/recreational variables predict positive and negative mental health similarly. In a representative sample of older Irish adults, active relational and recreational lifestyles, as well as being well integrated in social networks, were inversely related to later development of depression, anxiety and cognitive impairment.^20^ In a subsequent analysis using the same sample, these same predictors were found to be positively associated with continuous/ordinal measures of quality of life and self-rated mental health.^19^

Uncovering evidence to support the concept of differential predictors of low and high mental well-being is important for informing effective public health practice and the way interventions are designed. If socioeconomic predictors of high mental well-being do not mirror those of low mental well-being and mental disorders, existing mental health programmes relying on prevailing beliefs relating to social inequalities (whilst remaining pertinent for the prevention of mental illness) may not be sufficient or appropriate as a means to enhancing mental well-being.^16^

In this study, utilizing a large sample representative of the adult Danish population, we investigate socioeconomic and relational/recreational predictors of high and low mental well-being. This data set enabled (i) a direct comparison of predictors of low and high mental well-being, and (ii) a direct comparison of two related but different outcomes: low mental well-being and CMDs. Based on the aforementioned evidence, we hypothesized that (i) low socioeconomic position would be significantly associated with increased odds of low mental well-being and the presence of CMDs—but not significantly associated with high mental well-being, and (ii) relational/recreational behaviours would be significantly associated with increased odds of high mental well-being as well as reduced odds of low mental well-being and CMDs.

## Methods

### Study population

Data stem from the Danish Mental Health and Well-being Survey 2016,^27^ which is a representative sample of Danish men and women aged 16 years and above. Statistics Denmark sent an electronic letter to the sampled individuals in October 2016 with information about the study and an invitation to participate. A total of 1656 men and 1852 women responded to the web-based survey resulting in a response rate of 34%. Additionally, the survey has been linked up to the Danish civil registration system^28^ via Statistics Denmark, which includes information pertaining to education, employment status, income, etc. Each resident in Denmark has a personal registration number, enabling almost complete linkage between different registers.^29^ All data are anonymized and encrypted, so it cannot be traced back to specific participants.

### Mental well-being

The Warwick-Edinburgh Mental Well-being Scale (WEMWBS) is a well validated measure used to monitor mental well-being amongst a population, and is based on the conceptualization of mental well-being as feeling good and functioning well. The scale has recently been validated in Denmark.^4^ WEMWBS consists of 14 positively worded questions (shown in Supplementary appendix S1), leading to a score between 14 and 70; the higher the score, the higher mental well-being. We used a similar approach to Stewart-Brown et al.^16^ to define three population groups based on the continuous WEMWBS scale: the top 15th percentile (≈ 1SD above the mean and higher) with a WEMWBS score >60 defined as high mental well-being, the bottom 15th percentile (≈ 1SD below the mean and lower) with a WEMWBS score ≤43 defined as low mental well-being, and the remainder 16th–84th percentile with a WEMWBS score >43 and ≤60 defined as moderate mental well-being.

### Common mental disorders

CMDs were assessed with the Patient Health Questionnaire for Depression and Anxiety (PHQ-4).^30^ The PHQ-4 was developed to screen for core symptoms of depression and anxiety and has been validated with satisfactory sensitivity and specificity in terms of capturing anxiety and depressive disorders.^31^ The total scale ranges from 0 to 12, with higher scores indicating more symptoms of depression and anxiety. The optimal cut-point for each scale in the PHQ-4 is ≥3, which suggests clinically significant depression or anxiety.^31^ This cut-point was applied in this study, and CMD was operationalized as screening positive for depression and/or anxiety (≥3).

### Socioeconomic variables

All socioeconomic variables were extracted from the Danish civil registration system. Socioeconomic variables used for analysis were as follows: education (primary or unknown; youth education; tertiary education—low to high), marital status (married or registered partnership; divorced, terminated partnership or widowed; single), income (lowest quartile; second-lowest quartile; second-highest quartile; highest quartile), employment status (employed; unemployed; student; retired; early retirement; other—employment status not defined) and migration background (Danish ; immigrant or descendent of immigrant). Additionally, basic demographics were included for adjustment: sex (female; male) and age (continuous).

### Relational/recreational behaviours

Relational/recreational behaviour variables pertained to social interaction, social support, informal and formal social participation, and recreational activity, and were as follows: social interaction included seeing family, friends, colleagues (no or seldom; monthly); social support included having someone to rely on for social support (no or seldom; yes, often); informal social participation included helping others (no or seldom; monthly); formal social participation included volunteering (no or seldom; monthly); or being an active member of a community/social group (no or seldom; monthly). Finally, recreational activity involved engaging in a challenging activity or hobby (no or seldom; monthly).

### Statistical analysis

We used multinomial logistic regression in order to identify differences in the pattern of associations of socioeconomic, relational/recreational behaviour variables (predictors) with high and low mental well-being (outcomes), respectively, relative to moderate mental well-being (reference group). Binary logistic regression was used to identify differences in the pattern of associations of socioeconomic, relational/recreational behaviour variables (predictors) with the presence of CMDs (outcome). Thus, we built two models. In both models, we adjusted for sex (categorical) and age (continuous). A survey non-response statistical weight based on age, education, region, marital status, employment status and migration background was taken into account to attenuate selection bias. Both models were based on the sample with no missing data, and missing data for the sample (*N*=3508) were as follows: mental well-being 174 (5.0%); CMD 14 (0.4%); sex 0 (0%); age 10 (0.3%); marital status 10 (0.3%); migration background 0 (0%); education 0 (0%); income 238 (6.8%); employment status 7 (0.2%); seeing family, friends, colleagues 6 (0.2%); helping others 14 (0.4%); volunteering 16 (0.5%); community/social group membership 45 (1.3%); social support 3 (0.09%); and challenging activity/hobby 53 (1.5%).

## Results

The mean age in the study population was 52.1 years, and 52.8% of the participants were female (for more information on the demographic distribution see table 1). In the two adjusted models for mental well-being described below, moderate mental well-being was used as the reference group when estimating the associations between the predictors and each outcome. Due to the pattern in our results, we describe first the predictors of low mental well-being, then CMDs, and finally high mental well-being. The adjusted results are described below. Unadjusted univariate associations can be found in Supplementary appendix S2.

**Table 1:** Characteristics of the study sample

Characteristic	Category	*n*	%	% (weighted)
Total number of respondents (*N*)		3508	100	100
Sex	Female	1852	52.8	54.2
	Male	1656	47.2	45.8
Age (years)	Mean (SD)	47.0 (18.7)		
Education	Primary or unknown	831	23.7	33.9
	Gymnasium/youth education	1457	41.5	39.2
	Tertiary education	1220	34.8	26.9
Marital status	Married/registered partnership	1992	57.0	45.7
	Divorced, separated partners, widow	589	16.8	17.3
	Unmarried	917	26.2	37.1
Income	Highest quartile	817	23.3	30.9
	Second-highest quartile	818	23.3	26.5
	Third-highest quartile	818	23.3	23.1
	Lowest quartile	817	23.3	19.5
Employment status	Employed	1906	54.4	51.0
	Unemployed	147	4.2	5.1
	In work education	312	8.9	15.1
	Social pension/early retirement	948	27.1	21.8
	Early retirement	120	3.4	3.6
	Other	68	1.9	3.4
Migration background	Danish	3272	93.3	87.4
	Immigrant/descendent	236	6.7	12.6
Seeing family, friends, colleagues	No/seldom	414	11.8	12.5
	Yes, monthly	3088	88.2	87.5
Helps others	No/seldom	1713	49.0	47.5
	Yes, monthly	1781	51.0	52.5
Volunteering	No/seldom	2834	81.2	82.0
	Yes, monthly	658	18.8	18.0
Active membership in a community/social group	No/seldom	1818	52.5	57.0
	Yes, monthly	1645	47.5	43.0
Someone to rely on for social support	No/seldom	886	25.3	26.9
	Yes, often	2619	74.7	73.1
Engaging in challenging activity/hobby	No/seldom	1958	56.7	55.7
	Yes, monthly	1497	43.3	44.3
Mental well-being	Low	456	13.7	15.8
	Moderate	2364	70.9	69.2
	High	514	15.4	15.0
Common mental disorder	Present	878	25.1	27.9

Note: All data are *n* (%) unless otherwise specified.

### Low mental well-being

In the adjusted model (table 2), having a youth education, primary/unknown education status showed increased odds of having low mental well-being compared to tertiary education. Income in the lowest and second-lowest quartile showed increased odds for having low mental well-being relative to high income. Being unemployed or early retired both showed increased odds for low mental well-being, relative to being employed. Migration background did not show any association with having low mental well-being. Being divorced or not married were significantly associated with increased odds of having low mental well-being, relative to being married. All the relational/recreational behaviour variables were inversely associated with low mental well-being.

**Table 2:** Odds ratios for high and low mental well-being and CMD

	**Multinomial logistic regression** ^a^ **—adjusted for sex and age**				Binary logistic regression—adjusted for sex and age	
	High mental well-being		Low mental well-being		Common mental disorder	
	OR	95% CI	OR	95% CI	OR	95% CI
Marital status						
Married	Ref		Ref		Ref	
Divorced	1.11	0.84, 1.47	1.70*	1.24, 2.32	1.43*	1.14, 1.80
Not married	0.98	0.73, 1.31	2.09*	1.53, 2.84	1.20	0.95, 1.51
Migration background						
Danish	Ref		Ref		Ref	
Immigrant or descendant of immigrant	1.09	0.72, 1.65	1.06	0.70, 1.59	1.35	0.99, 1.84
Education						
Tertiary education	Ref		Ref		Ref	
Youth education	1.00	0.78, 1.27	1.60*	1.23, 2.09	1.74*	1.42, 2.14
Primary or unknown	1.10	0.84, 1.44	1.62*	1.21, 2.17	1.99*	1.59, 2.49
Income						
Highest quartile	Ref		Ref		Ref	
Second-highest quartile	0.78	0.58, 1.05	1.21	0.81, 1.79	1.16	0.88, 1.53
Second-lowest quartile	0.87	0.64, 1.18	2.37*	1.65, 3.39	1.72*	1.32, 2.24
Lowest quartile	0.77	0.57, 1.06	3.27*	2.31, 4.64	2.15*	1.66, 2.78
Employment status						
Employed	Ref		Ref		Ref	
Unemployed	0.56	0.26, 1.19	3.85*	2.54, 5.82	3.25*	2.23, 4.73
Student	1.34	0.84, 2.16	1.49	0.96, 2.32	1.28	0.90, 1.83
Retired	1.25	0.88, 1.76	1.13	0.77, 1.65	1.37*	1.03, 1.83
Early retirement	0.68	0.32, 1.41	4.66*	2.91, 7.46	3.09*	2.04, 4.70
Other (employment status not defined)	0.70	0.27, 1.84	1.74	0.89, 3.38	1.50	0.85, 2.64
Seeing family, friends, colleagues						
No/seldom	Ref		Ref		Ref	
Monthly	2.80*	1.69, 4.63	0.38*	0.29, 0.51	0.47*	0.36, 0.60
Someone to rely on for social support						
No/seldom	Ref		Ref		Ref	
Yes, often	3.52*	2.47, 5.01	0.24*	0.19, 0.31	0.43*	0.36, 0.52
Helping others						
No/seldom	Ref		Ref		Ref	
Monthly	1.55*	1.25, 1.92	0.69*	0.55, 0.86	0.86	0.72, 1.02
Volunteering						
No/seldom	Ref		Ref		Ref	
Monthly	1.49*	1.17, 1.91	0.64*	0.46, 0.90	0.79*	0.63, 0.99
Active member in a community/social group						
No/seldom	Ref		Ref		Ref	
Monthly	1.39*	1.12, 1.73	0.44*	0.35, 0.57	0.58*	0.49, 0.69
Engaging in challenging activity/hobby						
No/seldom	Ref		Ref		Ref	
Monthly	1.55*	1.24, 1.95	0.47*	0.37, 0.61	0.76*	0.63, 0.91

a Estimates for outcomes on high and low mental well-being were made relative to moderate mental well-being as part of the same multinomial regression model.

* *P*<0.05.

### Common mental disorders

The results for the associations between the socioeconomic, relational/recreational behaviour variables and the odds of having a CMD resembles the associations between the variables and the odds of having low mental well-being relative to moderate mental well-being (table 2). The pattern of point estimates for income and education showed increased odds of having a CMD among those with lower income and education. Being unemployed or early retired were associated with increased odds of a CMD, relative to being in employment. Those who were retired also had greater odds of having a CMD compared to those who were employed. Being divorced showed increased odds of having a CMD, compared with being married, while the association for non-married did not reach statistical significance. Migration status was not associated with CMDs.

Almost all of the relational/recreational behaviour variables showed significant negative associations with having a CMD: seeing family, friends, colleagues, volunteering and being an active member of a community/social group, having someone to rely on for social support, engaging in challenging activities/hobbies, were inversely associated with having a CMD. Helping others did not show a significant association with the odds of having a CMD.

### High mental well-being

In contrast to both low mental well-being and CMDs, none of the socioeconomic variables showed a statistically significant relationship with high mental well-being (table 2). Considering the coefficients regardless of significance testing, there was a pattern of reduced odds of high mental well-being for those with lower income and those with more unfavourable employment status (unemployed, early retired and other), but no pattern in terms of lower education. On the other hand, students and retired individuals showed increased odds of high mental well-being. All of the relational/recreational behaviour variables were positively associated with high mental well-being.

### Sensitivity analysis

As a sensitivity analysis, we conducted a linear regression with the continuous PHQ-4 scale as the outcome (results available upon request). The results were similar to those presented for CMD in table 2, but four variables reached statistical significance. These were (i) non-married, (ii) immigrant or descendant of immigrant, (iii) employment status—other and (iv) helping others. For both models, we also conducted an analysis where most predictors (education, income and all relational/recreational behaviour variables) were entered as continuous rather than categorical variables (results available upon request). Doing this resulted in the same overall patterns as the patterns reflected in table 2.

## Discussion

### Main findings

Socioeconomic predictors of low and high mental well-being do not mirror each other; socioeconomic factors are strongly associated with low mental well-being in a similar way to their association with CMDs, but they do not show a similar strong association with high mental well-being, i.e. they do not mirror each other. These findings support previous literature, that is, Stewart-Brown et al.,^16^ Van Lente et al.,^18^ Nielsen et al.^17^ and Solin et al.^21^ arrived at similar results as the current study in widely differing populations. While this pattern is evident in the case of the socioeconomic predictors, relational/recreational behaviours are associated with both low mental well-being and CMDs (negatively) as well as high mental well-being (positively).

### Limitations

Some limitations should be taken into account when interpreting the results. The study design is cross-sectional and so does not establish causality, and the data unfortunately did not contain variables that could have been instrumental in assessing the direction of associations (e.g. retrospective data). Future studies are warranted to conduct similar analyses with longitudinal data. Further, the response rate was 34% and while this is not unusual for web-based surveys, selection bias cannot be ruled out. Response rates were higher for women, individuals aged 45 years old and above, individuals with higher education (tertiary), individuals who were married or in a registered partnership, employed individuals, individuals with a Danish (non-migrant) background and individuals with higher income.^27^

### Contextualization of findings

Our results support the notion that reducing low socioeconomic position is valuable in preventing poor mental health (i.e. low mental well-being and CMDs), given that low income, education, employment status, finance and the lack of a significant other may involve considerable distress to an individual.^14^ Thus, focusing efforts on improving socioeconomic conditions is warranted when the desired outcome is to prevent poor mental health. However, we do not find evidence that improving socioeconomic conditions would in and of itself bring about higher levels mental well-being, such as would be required in a population or ‘shifting the curve’ approach to mental health (i.e. shifting the entire population towards higher levels of mental well-being).^32^

Our results support a ‘hierarchy of needs’ theoretical viewpoint where material conditions predict well-being up to a certain point but become less influential as material needs are satisfied and dominant needs shift upward.^14^ Although our results did indicate a pattern of reduced odds of high well-being for lower education and more unfavourable employment status, these results did not reach statistical significance as they did with low mental well-being and CMDs. This suggests that the associations are not as robust, and while these socioeconomic factors may not be entirely irrelevant in regards to high mental well-being, our results indicate that other factors, such as social support, take priority. Relational/recreational behaviours appear to be relevant at both ends of the mental health spectrum, i.e. both low mental well-being and CMDs as well as high mental well-being. In other words, if the desired outcome is to promote mental well-being across an entire population regardless of mental health status or risk, salutogenic strategies focusing on relational/recreational behaviours, such as social interaction, participation and engagement in meaningful activities, may be essential.^33^^,^^34^

It is of interest that the variable ‘married/divorced/not married’ behaved in the same way as socio-economic variables like income, education and employment rather than the relational/recreational behaviour variables, in that it was associated with low mental well-being and CMDs, but not with high mental well-being. Living alone has implications for both social support and social networks which are both strongly related to high mental well-being. On the other hand, marital status and divorce both have implications for income, which may explain why the variable behaves more in line with socio-economic variables.

### Implications for policy and practice

The results have implications in public health policy and practice because they suggest that different strategies may be necessary, depending on whether they aim to prevent low mental well-being and CMDs, or promote high mental well-being. Two distinct but complementary strategies exist in the field of disease prevention and health promotion: universal and specific.^34^ The *specific* strategy seeks to target particular sub-groups (often at-risk groups) in a population, while the *universal* strategy has the entire or parts of the population as target group, regardless of risk factors or behaviours. In the light of the results obtained in this study, efforts to address low socioeconomic position would be particularly relevant in specific prevention strategies focusing on low mental well-being and CMDs, but less relevant in terms of promoting high levels of mental well-being. The results further suggest that relational/recreational factors may be universally relevant whether the desired outcome is the prevention of low mental well-being and CMDs or the promotion of high levels of mental well-being. Research has demonstrated that the relationship between social connectedness/activity and mental health is bi-directional, i.e. social connectedness and activity enhances mental health, and conversely, people with better mental health are also more likely to be socially connected and engage in social activity.^35^ Such findings are in line with increasing recognitions that mental illness is both a cause and a consequence of social inequities, i.e. mental illness reflects both a deprivation in socially protective factors and also contributes to the lack of them.^36^

The wider implications of our results are that rather than just focusing on what drives and protects against mental disorders, governments must also consider the drivers of positive mental health,^37^ and prioritize them accordingly.^2^ Promoting mental health and well-being throughout a population can mean encouraging active lifestyles,^38^ providing opportunities for people to interact and feel they belong within a community,^39^ and fostering a sense of purpose by advocating contribution to society or engagement in meaningful activities and causes.^33^ Strategies that have shown potential in the general population as well as sub-populations pertaining to mental disorders include those highlighted in the Act-Belong-Commit programme also referred to as the ABC’s of mental health.^40^ Strategies may also include efforts focused on individuals, such as encouraging self-care and opportunities to hone personal and social skills and pursue creative endeavours. The combination of universal and individual approaches has proved important in many different settings.^5^

## Conclusion

This study has demonstrated that the socioeconomic predictors of high mental well-being do not mirror those of low mental well-being and CMDs, whereas relational/recreational behaviours associated with high mental well-being do mirror those of low mental well-being and CMDs. Specific mental health strategies addressing socioeconomic factors are valuable and necessary in regard to preventing poor mental health but may be limited in universal approaches and those addressing the high end of the mental health spectrum. Strategies focusing on relational/recreational behaviours may be essential in both preventing poor mental health as well as promoting higher levels of mental well-being. In order to increase the likelihood of implementing successful strategies, universal, holistic and comprehensive approaches are needed to address all levels of mental well-being throughout a population.

## Supplementary data

Supplementary data are available at *EURPUB* online.

## Disclaimer

This study was funded by the Nordea-fonden. The funder of the study had no role in study design, data collection, data analysis, data interpretation or the writing of the report. The corresponding author had full access to all of the data in the study and the final responsibility to submit for publication.

*Conflicts of interest*: None declared.

## Supplementary Material

ckaa021_Supplementary_Data
