# Supplementary material for: Predictors of high and low mental well-being and common mental disorders: findings from a Danish population-based study
Source: Eur J Public Health. 2020 Feb 27;30(3):503–9. doi: 10.1093/eurpub/ckaa021 (PMC7292340; doi:10.1093/eurpub/ckaa021)
Supplement: ckaa021_Supplementary_Data [file ckaa021_supplementary_data.zip › ejph-2019-11-om-0894-File004.docx]

**Appendix 1**

| Items included in the WEMWBS questionnaire^1^: |
| --- |
| 1. I've been feeling optimistic about the future. |
| 1. I've been feeling useful. |
| 1. I've been feeling relaxed. |
| 1. I've been feeling interested in other people. |
| 1. I've had energy to spare. |
| 1. I've been dealing with problems well. |
| 1. I've been thinking clearly. |
| 1. I've been feeling good about myself. |
| 1. I've been feeling close to other people. |
| 1. I've been feeling confident. |
| 1. I've been able to make up my own mind about things. |
| 1. I've been feeling loved. |
| 1. I've been interested in new things. |
| 1. I've been feeling cheerful. |
| ^1^Answers options were: “none of the time”, “rarely”, “some of the time”, “often”, “all of the time”. |

Warwick-Edinburgh Mental Well-being Scale (WEMWBS) © NHS Health Scotland, University of Warwick and University of Edinburgh, 2006, all rights reserved
