# Supplementary material for: Predictors of high and low mental well-being and common mental disorders: findings from a Danish population-based study
Source: Eur J Public Health. 2020 Feb 27;30(3):503–9. doi: 10.1093/eurpub/ckaa021 (PMC7292340; doi:10.1093/eurpub/ckaa021)
Supplement: ckaa021_Supplementary_Data [file ckaa021_supplementary_data.zip › ejph-2019-11-om-0894-File005.docx]

| **Appendix 2.** Odds ratios for high and low mental well-being and common mental disorder | | | | | | |
| --- | --- | --- | --- | --- | --- | --- |
|  | Multinomial logistic regression^a^ –  Univariate | | | | Binary logistic regression – Univariate | |
|  | High mental well-being | | Low mental well-being | | Common mental disorder | |
|  | OR | 95% CI | OR | 95% CI | OR | 95% CI |
| Marital status |  |  |  |  |  |  |
| Married | Ref |  | Ref |  | Ref |  |
| Divorced | 1.15 | 0.87, 1.52 | 1.74* | 1.29, 2.36 | 1.38* | 1.11, 1.73 |
| Not married | 0.87 | 0.67, 1.12 | 2.26* | 1.77, 2.89 | 1.55* | 1.29, 1.89 |
| Migration background |  |  |  |  |  |  |
| Danish | Ref |  | Ref |  | Ref |  |
| Immigrant or descendant of immigrant | 0.98 | 0.65, 1.48 | 1.13 | 0.76, 1.69 | 1.45* | 1.08, 1.95 |
| Education |  |  |  |  |  |  |
| Tertiary education | Ref |  | Ref |  | Ref |  |
| Youth education | 1.02 | 0.80, 1.30 | 1.56* | 1.20, 2.03 | 1.65* | 1.34, 2.02 |
| Primary or unknown | 1.06 | 0.81, 1.40 | 1.68* | 1.26, 2.24 | 1.99* | 1.60, 2.49 |
| Income |  |  |  |  |  |  |
| Highest quartile | Ref |  | Ref |  | Ref |  |
| Second highest quartile | 0.76 | 0.56, 1.02 | 1.25 | 0.84, 1.86 | 1.20 | 0.91, 1.58 |
| Second lowest quartile | 0.85 | 0.63, 1.15 | 2.45* | 1.71, 3.51 | 1.77* | 1.36, 2.30 |
| Lowest quartile | 0.76 | 0.56, 1.04 | 3.46* | 2.44, 4.90 | 2.28* | 1.76, 2.95 |
| Employment status |  |  |  |  |  |  |
| Employed | Ref |  | Ref |  | Ref |  |
| Unemployed | 0.55 | 0.26, 1.17 | 4.08* | 2.70, 6.17 | 3.47* | 2.41, 5.03 |
| Student | 1.18 | 0.83, 1.70 | 1.84* | 1.31, 2.60 | 1.75* | 1.34, 2.30 |
| Retired | 1.42* | 1.13, 1.80 | 0.91 | 0.68, 1.22 | 1.00 | 0.82, 1.23 |
| Early retirement | 0.69 | 0.32, 1.48 | 4.54* | 2.85, 7.22 | 2.93* | 1.94, 4.43 |
| Other (employment status not defined) | 0.65 | 0.25, 1.69 | 2.06* | 1.08, 3.94 | 1.91* | 1.10, 3.32 |
| Seeing family, friends, colleagues |  |  |  |  |  |  |
| No/seldom | Ref |  | Ref |  | Ref |  |
| Monthly | 2.80* | 1.69, 4.62 | 0.42* | 0.32, 0.55 | 0.51* | 0.40, 0.65 |
| Someone to rely on for social support |  |  |  |  |  |  |
| No/seldom | Ref |  | Ref |  | Ref |  |
| Yes, often | 3.31* | 2.32, 4.71 | 0.26* | 0.20, 0.32 | 0.46* | 0.38, 0.56 |
| Helping others |  |  |  |  |  |  |
| No/seldom | Ref |  | Ref |  | Ref |  |
| Monthly | 1.48* | 1.19, 1.84 | 0.75* | 0.60, 0.93 | 0.89 | 0.74, 1.05 |
| Volunteering |  |  |  |  |  |  |
| No/seldom | Ref |  | Ref |  | Ref |  |
| Monthly | 1.50* | 1.17, 1.92 | 0.63* | 0.44, 0.88 | 0.77* | 0.61, 0.94 |
| Active member in a community/social group |  |  |  |  |  |  |
| No/seldom | Ref |  | Ref |  | Ref |  |
| Monthly | 1.44* | 1.17, 1.80 | 0.41* | 0.32, 0.53 | 0.55* | 0.46, 0.66 |
| Engaging in challenging activity/hobby |  |  |  |  |  |  |
| No/seldom | Ref |  | Ref |  | Ref |  |
| Monthly | 1.44* | 1.16, 1.79 | 0.53* | 0.42, 0.68 | 0.82* | 0.69, 0.97 |
| * p < 0.05  ^a^ Estimates for outcomes on high and low mental well-being were made relative to moderate mental well-being as part of the same multinomial regression model | | | | | | |
